# E-cigarette, or Vaping, Product Use-associated Lung Injury (EVALI): Acute Lung Illness within Hours of Switching from Traditional to E-cigarettes

**DOI:** 10.7759/cureus.7513

**Published:** 2020-04-02

**Authors:** Smit Deliwala, Saira Sundus, Tarek Haykal, Nikita Theophilus, Ghassan Bachuwa

**Affiliations:** 1 Internal Medicine, Hurley Medical Center, Michigan State University, College of Human Medicine, Flint, USA; 2 Internal Medicine, Hurley Medical Center, Michigan State University, Flint, USA

**Keywords:** e-cigarette and vaping product use associated lung injury (evali), evali, vaping, electronic cigarette associated lung injury, e-cigarettes, electronic cigarette, acute lung injury, lung injury, chest ct, chest radiograph

## Abstract

2019 has been a landmark year in the world of electronic nicotine delivery systems (ENDS), specifically e-cigarette and vaping. Numerous state health departments across the United States have voiced their concerns in the growing number of lung injury cases from e-cigarettes and vaping. Over the past few decades, many agencies have brought into light the harmful effects of smoking cigarettes, and despite popular belief, a growing movement has started to recognize the harmful effects of ENDS. The Centers for Disease Control and Prevention have released recommendations and provided health practitioners a methodology to identify and diagnose e-cigarette, or vaping, product use-associated lung injury (EVALI). EVALI is a diagnosis of exclusion and comprises a variety of respiratory illnesses, with intubation rates nearing 32%. The most critical risk factor remains product use in the preceding 90 days, although a timeline on the development of symptoms or notable structural changes remains unknown. We present a case of acute lung injury in a traditional cigarette smoker that evolved within hours of switching to e-cigarettes.

## Introduction

The 2019 national outbreak of e-cigarette, or vaping, product use-associated lung injury (EVALI) reported by 25 state health departments included hundreds of reported cases. By January 2020, over 2000 cases had been reported to the Centers for Disease Control and Prevention (CDC) [1]. The epidemic set the stage for the CDC to release interim recommendations to health care providers to recognize and manage respiratory problems arising from electronic nicotine delivery systems (ENDS) [2,3]. ENDS comprises electronic cigarettes, pipes, vaporizers, cigars, and numerous other products. EVALI is an umbrella term incorporating a spectrum of respiratory illnesses ranging from mild cases of pneumonitis to life-threatening lung failure with the most extensive case series reporting 32% of patients requiring intubation and mechanical ventilation [4]. Reported trends reveal 80% of users to be between 13 to 85 years of age, while most users were male [2,3]. Analysis of these products has not revealed any evidence of tainting with microbes, and the constellation of symptoms seems to be more in line with acute lung injury [5]. The use of electronic cigarettes or the equivalent within the past 90 days continues to be the primary risk factor. A review of the literature reveals various types of injuries from the use of ENDS, such as explosion injuries, dermal reactions, while the most common remains the development of respiratory symptoms. Despite substantial reductions in usage, incidence rates have continued to progress, placing precedence on continued surveillance and public outreach efforts. Although advancements in researching e-cigarettes have grown, its instantaneous and prolonged influences on health remain unclear. Reports focusing on symptom timelines are scarce, ranging as early as few days after initial use to the development of symptoms after months or years of chronic smoking. We present a case of acute lung injury that manifested within hours of first-time e-cigarette use. This report focuses on the duration and timeline of symptom development with an emphasis on acute lung injuries that manifested within one month of ENDS product use.

## Case presentation

A 41-year-old man presented to an urgent care facility with an unrelenting cough and shortness of breath that began after multiple uses of an e-cigarette within the span of a few hours. He had been a daily cigarette smoker for 11 years, had never used electronic methods of smoking before and decided to transition to e-cigarettes with the plan to eventually quit smoking. He had acquired the unlicensed product from a friend, with the primary ingredient being a nicotine-based oil. He was diagnosed with tracheobronchitis after a negative chest radiograph, and was given a five-day course of azithromycin and requested to follow up with his primary care physician (PCP). Despite the antibiotic, his symptoms evolved over the next 48 hours to nausea, vomiting, pleuritic chest and abdominal pain, non-productive cough, and worsening shortness of breath, prompting him to go to the emergency department (ED). He denied having been diagnosed with any medical conditions, including symptoms consistent with episodes of bronchitis, pneumonia, or an admission that required supplemental oxygen in the past. He denied experiencing similar symptoms or adverse reactions to smoking in the past, nor did he have occupational or domestic inhalation exposures, recent travel, or exposures to sick contacts. On admission, blood pressure was 186/108, heart rate was 141, O2 saturation was 98% on room air, tachypneic to 23, and afebrile. On exam, he appeared ill, coughing and was writhing in pain, while the remaining exam was unremarkable. He was given ketorolac for analgesia and 0.1 mg of clonidine for his elevated blood pressure. Empiric azithromycin 500 mg and ceftriaxone 2000 mg were given for community-acquired pneumonia (CAP). Interspersed were albuterol 2.5 mg and ipratropium 0.02% nebulization treatments every four hours for his symptoms.

**Figure 1:**
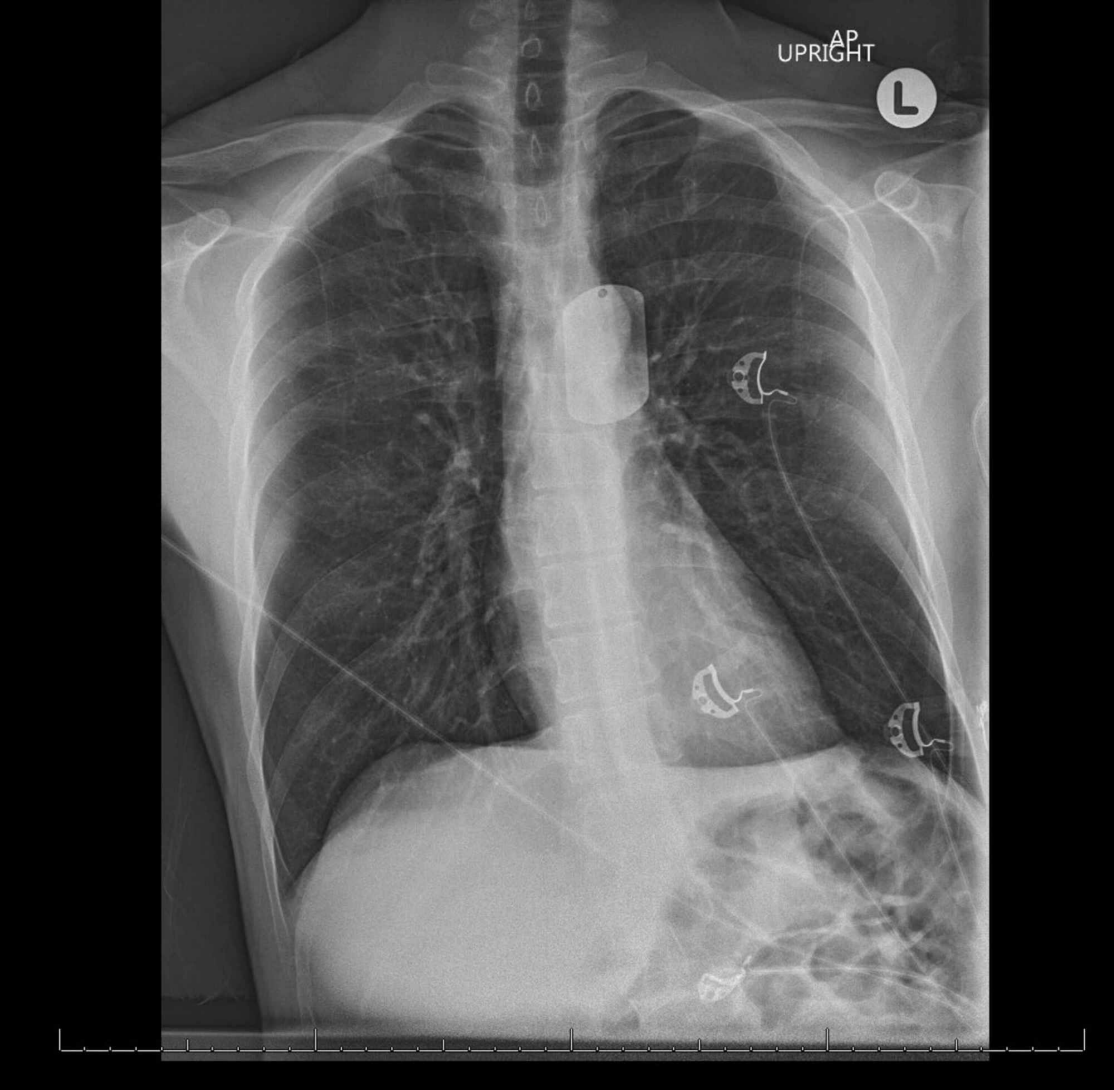
An unremarkable anteroposterior chest radiograph on admission

**Figure 2:**
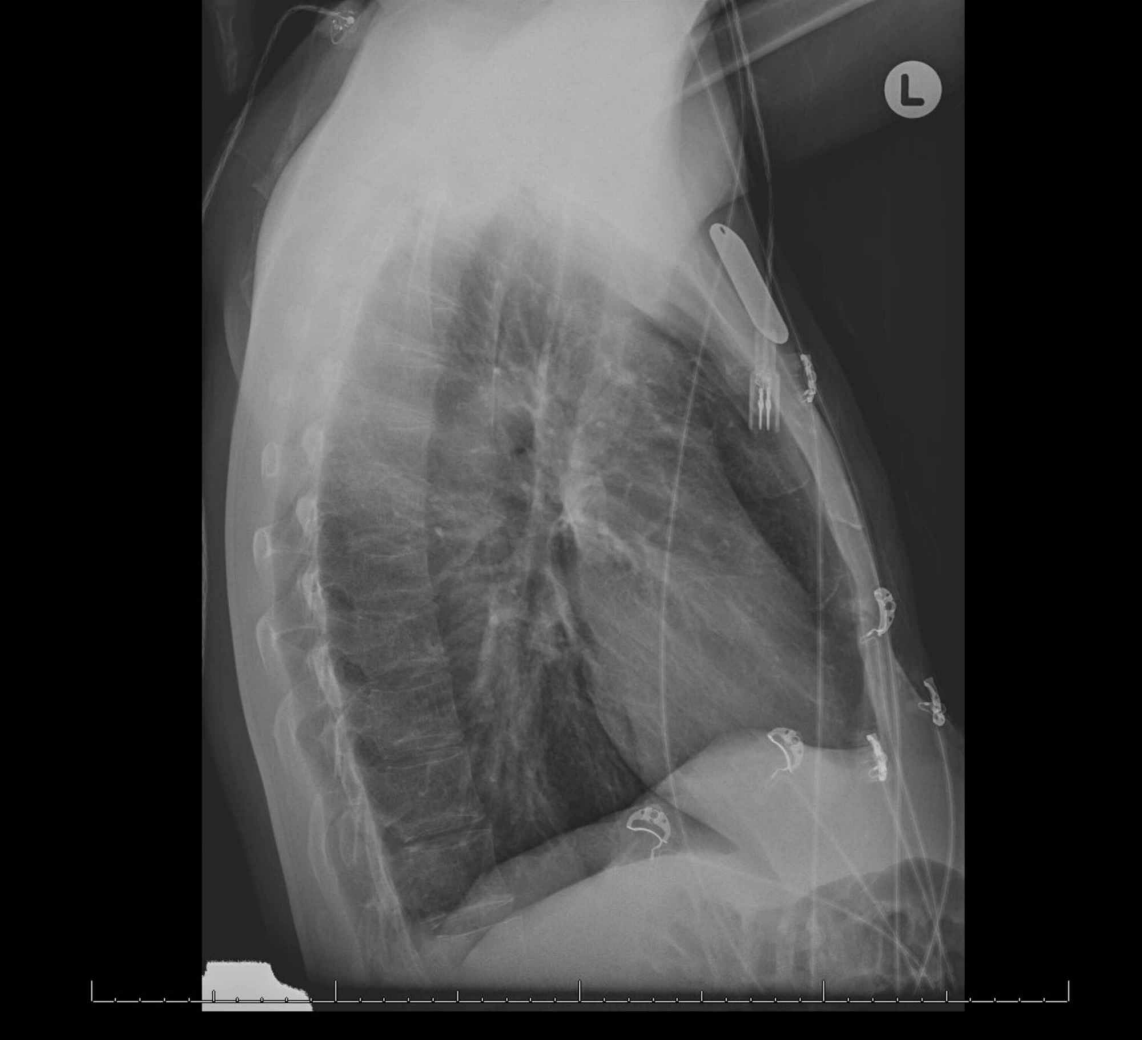
An unremarkable lateral chest radiograph on admission

**Figure 3:**
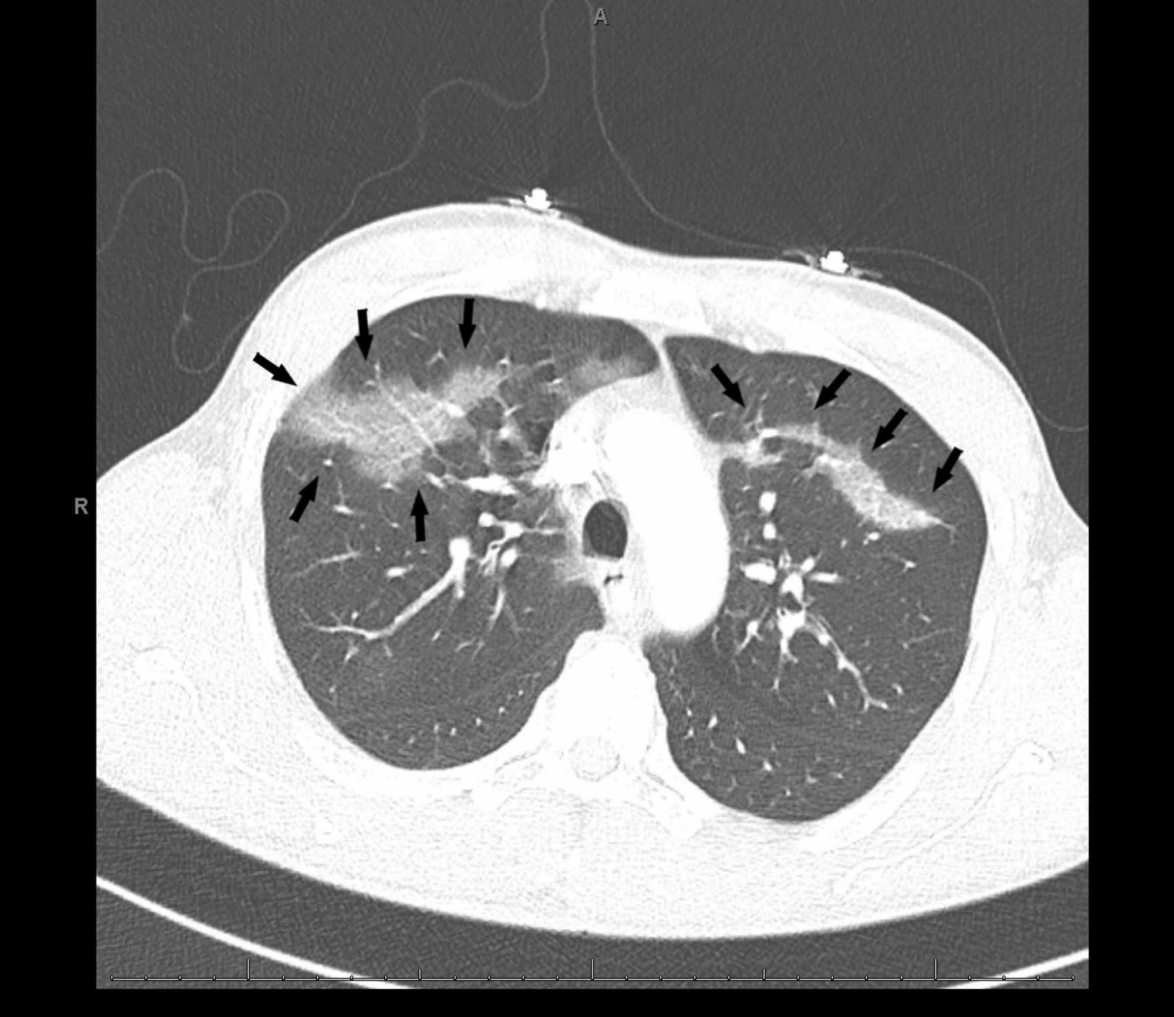
A computed tomography (CT) of the chest with bilateral ground-glass opacities (black arrows)

**Figure 4:**
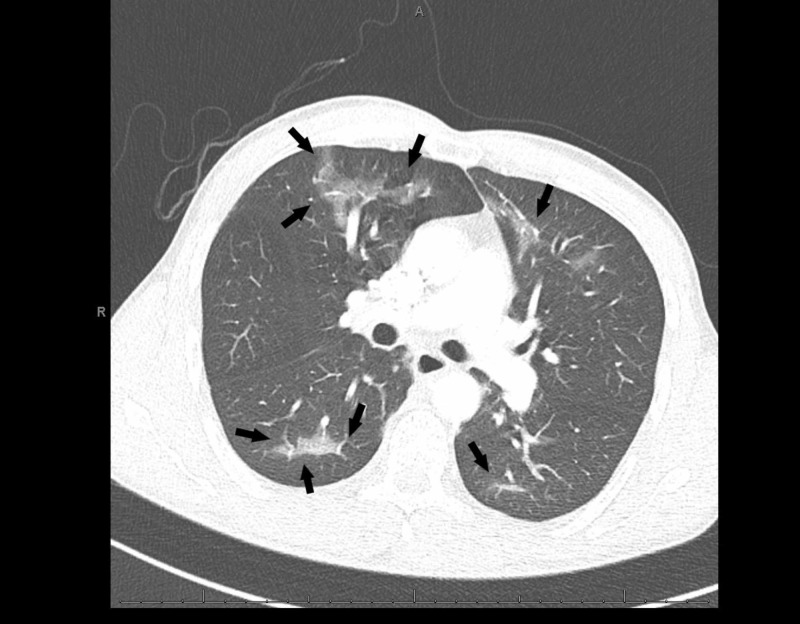
A computed tomography (CT) of the chest with bilateral ground-glass opacities seen with involvement of the posterior parenchyma (black arrows)

**Figure 5:**
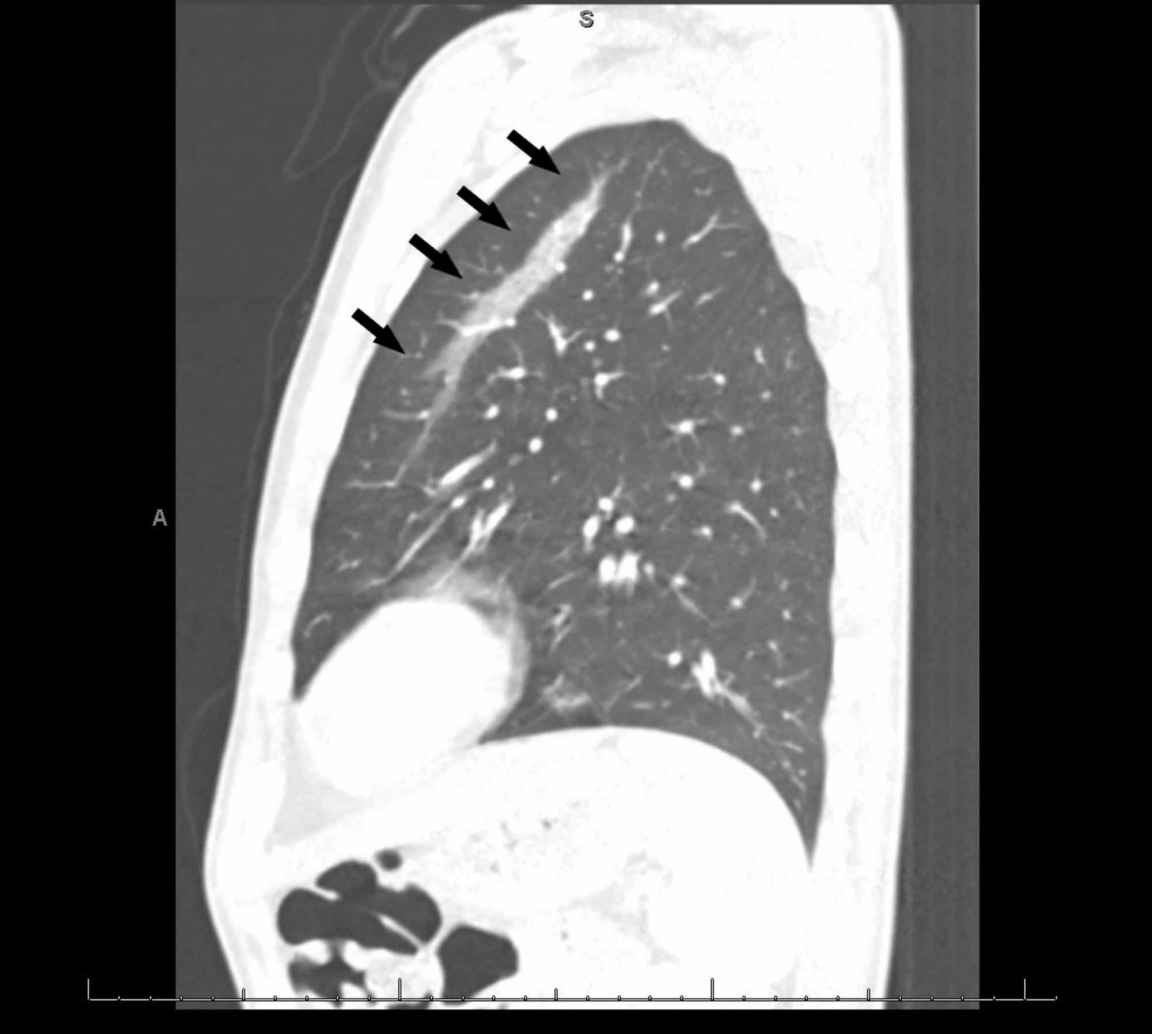
A lateral computed tomography (CT) of the chest revealing changes consistent with ground-glass opacities

His initial complaint of chest pain prompted an ECG and troponin testing that were unremarkable. Complete blood count was notable for neutrophilic leukocytosis of 12.1 K/UL, while serum chemistries were unremarkable. Antero-posterior and lateral chest X-rays were unremarkable (Figures 1, 2). However, his persistent tachypnea and shortness of breath with a moderate pretest probability prompted a computed tomography (CT) angiogram suspecting pulmonary embolism (PE). Imaging revealed parenchymal changes consistent with bilateral upper and lower lobe ground-glass opacities (Figures 3-5). No septal change was noted, helping us rule out causes such as organizing pneumonia, lipoid pneumonia, and diffuse alveolar damage. Extensive testing for viral and bacterial infections was all negative (Table 1). During his stay, he remained hemodynamically stable with an oxygen saturation above 92%, and his pain had improved. Bronchoalveolar lavage (BAL) testing was not considered due to the rapidity of clinical improvement. He was switched to levofloxacin 750 mg daily towards a full CAP course and recommended over the counter ibuprofen for his sore chest. At his two-week visit, he endorsed giving up e-cigarettes but had resumed traditional cigarette smoking, and denied experiencing any further soreness or pain.

**Table 1:** Laboratory investigations during electronic, or vaping, product use-associated lung injury (EVALI)

Laboratory investigation	Value
Sodium	133 meq/L
Potassium	4.0 meq/L
Magnesium	1.4 mg/dL
Creatinine	0.7 mg/dL
Anion Gap	8 meq/L
White Blood Cell Count	12.1 K/uL
Hemoglobin	13.4 g/dL
Platelet Count	269 K/uL
Neutrophil	83%
Lymphocyte	10%
Routine HIV Ag/Ab 4^th^ Generation	Negative
Hepatitis B Surface Antigen	Negative
Hepatitis B Surface Antibody	Negative
Hepatitis C Antibody	Negative
Toxicology Screen	Positive for Opiates & Tetrahydrocannabinol (THC)
Troponin I	<0.01 ng/ml
Urine Legionella antigen	Negative
Adenovirus	Negative
Coronavirus 229E	Negative
Coronavirus HKU1	Negative
Coronavirus NL63	Negative
Coronavirus OC43	Negative
Human metapneumovirus	Negative
Human Enterovirus/Rhinovirus	Negative
Influenza A	Negative
Influenza B	Negative
Parainfluenza Virus (PIV) 1	Negative
Parainfluenza Virus (PIV) 2	Negative
Parainfluenza Virus (PIV) 3	Negative
Parainfluenza Virus (PIV) 4	Negative
Respiratory Syncytial Virus	Negative
Bordetella parapertussis	Negative
Bordetella pertussis by Polymerase Chain Reaction (PCR)	Negative
Chlamydia Pneumonia	Negative
Mycoplasma Pneumonia	Negative
Streptococcus Pneumonia antigen	Negative
Sputum cultures	No growth

## Discussion

We present a case of acute lung injury using the EVALI diagnostic criteria recently proposed by the CDC [2,3]. Although the exact pathogenesis of EVALI is unknown, a recent analysis of 29 bronchoalveolar lavages (BAL) revealed vitamin E acetate in all the samples, making it the first concerning ingredient in these injuries [6]. Similarly, in a follow-up study of 51 BAL samples collected across the country, 94% of the non-healthy samples contained vitamin E acetate. Among other additives, tetrahydrocannabinol (THC) was noted in 75 to 90 percent of the samples, while isolated nicotine was observed in 13-58 percent [2,3]. One coordinated public health effort found 94% of patients using an ENDS product in the week preceding their exacerbation, demonstrating a crucial modifiable risk factor [5]. In a patient with product use, respiratory symptoms, a negative chest radiograph, and clinical suspicion of lung injury, a CT scan of the chest can provide diagnostic value [7]. Consider admission for patients with respiratory distress, O2 saturation below 95% on room air, or concern for lung injury from e-cigarettes used within the preceding 90 days, while many EVALI patients have had persistent hypoxemia requiring home oxygen and bronchodilators at discharge, long-term effects are unknown, and follow-up is strongly advised [3,8]. Despite the scarcity of reported short- and long-term adverse effects from these products, animal and observational studies and a few clinical trials have tried to fill this gap, finding numerous adverse effects from these products. Conditions such as hypersensitivity pneumonitis, lipoid pneumonia, and eosinophilic pneumonia have demonstrated oxidative stress, protease concentrations, and alveolar changes similar to the lungs of a traditional tobacco smoker [9].

The National Academies of Sciences, Engineering, and Medicine (NASEM), based on studies of various compounds found in these products, released statements to address the myths of ENDS [10]. It reported limited evidence for improvement in lung function and respiratory symptoms in adults who switched to e-cigarettes, and a more robust recommendation that ENDS does have respiratory effects and can increase coughing and wheezing, and possibly exacerbate underlying asthma [10]. Inhaled nicotine is linked to chronic airway disease and lung remodeling from phenotypic studies based on levels of cytotoxic damage from increased mitochondrial reactive oxygen species seen after aerosol exposure of nanoparticles leading to an unstable electron transport chain and nuclear DNA disintegration [11,12]. The most extensive case series of patients accrued from the Illinois and Wisconsin departments of health demonstrated a variety of symptomatology, with 98% of the patients presenting with respiratory symptoms, 81% with gastrointestinal symptoms, and almost all having constitutional symptoms. Imaging revealed bilateral infiltrates in all cases, while 94% of patients were hospitalized, and 32% required intubation and mechanical ventilation. Despite popular belief, a meta-analysis reviewing trends among smokers and ever smokers demonstrated that the probability of using e-cigarettes was much more significant in current smokers than ever smokers. At the same time, a subgroup analysis revealed that ever e-cigarette use was more commonly seen within adolescent smokers [13].

EVALI is a diagnosis of exclusion, and numerous other causes must be ruled out in the process of confirmation. However, the most critical risk factor remains e-cigarette or vaping use in the 90 days preceding symptom onset. Confirmed cases often include findings of opacities on chest radiography or CT and the absence of pulmonary infections, including viral and bacterial pathogens. Sputum cultures can be obtained while HIV testing for opportunistic infections should be completed as well [2]. A thorough literature review revealed multiple cases of EVALI with varying durations of product use before symptom development, ranging from a few hours to months. We consolidated cases where the reported duration was less than one month of product use in Table 2, while cases with unknown duration, or beyond one month were excluded. Our goal was to parse out cases with rapid onset, although this does not represent an exhaustive list. The importance of our case stems from its acute presentation and the possibility that e-cigarettes may pose a higher risk in existing smokers, as seen in our patient who never had any symptoms until the e-cigarette use. Our patient developed symptoms of chest and abdominal pain immediately after using the product for the first time. Only one other case reports such quick onset of symptoms, although not during the first time of use [14]. EVALI has far-reaching consequences, and avoidance of all products is paramount, in contrast, follow-up is crucial, as many can continue to experience respiratory symptoms with continued dependence on supplemental oxygen.

**Table 2:** Overview of reported respiratory cases from e-cigarette or vaping use less that fall under the electronic, or vaping-associated lung injury (EVALI) spectrum with duration of product use less than one month

Author	Number of cases	Duration of product use prior to diagnosis	Diagnosis	Chest X-ray	Chest CT	Bronchoalveolar lavage
He et al. [14]	1	6 hours	Severe acute lung injury	Not done	Extensive airspace opacification in a centrilobular nodular pattern	Hemorrhagic return
Hureaux et al. [15]	1	One week	Subacute bronchial toxicity	Unchanged	Not done	Not done
Thota and Latham [16]	1	3 days	Acute eosinophilic pneumonitis	Subtle diffuse patchy reticulonodular opacities	Diffuse ground glass opacities involving the upper and middle lobes of the lungs more than the lower lobes	White blood cells with eosinophilia
Moore et al. [17]	1	3 days	Acute bilateral pneumonia with bilateral pulmonary effusions	Bilateral hypo inflated lungs with bibasilar parenchymal consolidation and bilateral pleural effusions	Unremarkable	Not done
Mantilla et al. [18]	1	4 weeks	Bronchiolitis obliterans organizing pneumonia	Unknown	Bilateral multiple small pulmonary nodules	Unknown
Long et al. [19]	1	4 weeks	Inhalation injury leading to diffuse alveolar hemorrhage	Bilateral diffuse lung infiltrates	Bilateral diffuse lung infiltrates	Hemorrhagic return
Sommerfeld et al. [20]	1	2-3 weeks		Patchy bilateral pulmonary infiltrates	Bilateral opacities, interlobular septal thickening and bilateral pleural effusions	cellular debris and reactive mononuclear cells, and cell counts were remarkable for elevated mononuclear cells and eosinophilia

## Conclusions

E-cigarette, or vaping product use-associated lung injury (EVALI) is a spectrum of respiratory manifestations, with the most critical risk factor being e-cigarette or vaping in the 90 days preceding symptom onset. It is a diagnosis of exclusion requiring imaging modalities demonstrating bilateral changes, ruling out of viral, bacterial, and atypical pathogens and other pulmonary pathologies. The most common adulterants identified in samples are vitamin E acetate, THC, and nicotine. Symptoms may develop as early as a few hours after inhalation to months after use. Follow-up care is vital to demonstrate the resolution of symptoms and radiographic findings. The Centers for Disease Control and Prevention have released interim guidelines to identify and manage cases of EVALI. At the same time, more research is required to parse out the pathophysiology and mechanism behind lung injury and the instantaneous effects that these devices pose while long-term studies are essential.
